# Long-term remission in advanced stage hepatocellular carcinoma? A chance for cure?

**DOI:** 10.1007/s12254-018-0431-z

**Published:** 2018-08-24

**Authors:** Matthias Pinter, Wolfgang Sieghart

**Affiliations:** 1 Division of Gastroenterology & Hepatology, Department of Internal Medicine III, Medical University of Vienna, Waehringer Guertel 18–20, 1090 Vienna, Austria; 2 Liver Cancer (HCC) Study Group Vienna, Medical University of Vienna, Vienna, Austria; 3 Comprehensive Cancer Center Vienna, Medical University of Vienna, Vienna, Austria

**Keywords:** Targeted therapy, Sorafenib, Tyrosine kinase inhibitor, Immunotherapy, Complete response

## Abstract

Liver resection, transplantation, and local ablation are potential curative treatment options but can only be offered to patients with early stage hepatocellular carcinoma (HCC). Patients with macrovascular tumor invasion and extrahepatic metastases are candidates for palliative systemic therapies. Achieving radiological complete response can be associated with long-term remission and excellent outcome. However, despite recent advancements in the medical treatment of advanced stage HCC, complete remission with available systemic treatment options still remains a rare event. This review summarizes data on radiological complete response to systemic therapies and discusses issues that may complicate the goal of achieving cure in advanced stage HCC.

## Introduction

Hepatocellular carcinoma (HCC) is the most common primary liver cancer [1, 2] and the second most common cause of cancer-related death globally [3].

Potential curative therapies can only be offered to patients with early stage HCC (small tumors limited to the liver) and include liver resection, liver transplantation, and local ablative therapies (i.e., radiofrequency or microwave ablation; [4]). About one-third of all patients with newly diagnosed HCC are candidates for potential curative treatments, while the vast majority is still subject to palliative therapy. Of these, about 40% of patients present with advanced stage HCC [5], characterized by one or more of the following features: macrovascular invasion (MVI), extrahepatic metastases, or symptomatic tumors (performance status 1–2). These patients are classical candidates for systemic therapy [4]. Beside the reference standards sorafenib (first-line) and regorafenib (second-line) [4, 5], other targeted therapies have already succeeded in phase III trials—lenvatinib in first-line as well as cabozantinib and ramucirumab both in second-line [6–8]—and will shortly be added to the treatment armamentarium. Additionally, the PD-1 targeted antibody nivolumab was conditionally approved in sorafenib-experienced patients in the Unites States, based on promising data from a phase I/II study [9].

This review summarizes data on complete remission to available systemic treatment options and discusses issues that may complicate the goal of achieving cure in locally advanced or metastatic HCC.

## Definition of remission and cure

The National Cancer Institute (NCI) defines complete response (CR)/remission as the disappearance of all signs of cancer in response to treatment (https://www.cancer.gov/publications/dictionaries/cancer-terms?expand=C). Radiological response in solid cancers is usually assessed by the Response Evaluation Criteria in Solid Tumors (RECIST; [10]) and its revised version (RECIST 1.1; [11]), respectively. According to these guidelines, CR requires the disappearance of all target and non-target lesions as well as a normalization of tumor marker levels [10, 11]. RECIST is based on tumor shrinkage which is reasonable when evaluating response to cytotoxic chemotherapy. However, these criteria do not take into account tumor necrosis and may be misleading when applied to molecular targeted therapies in HCC. Therefore, the original RECIST were later amended to incorporate the concept of viable tumor tissue—proposed by the European Association for the Study of the Liver (EASL) and characterized by contrast enhancement of the lesion in the arterial phase of radiological imaging [12]—forming the modified RECIST (mRECIST) criteria for HCC [13]. Accordingly, CR is defined as the disappearance of any intratumoral arterial enhancement in target and non-target lesions [13]. The mRECIST criteria for HCC were later adopted by the European EASL guidelines on the management of HCC [4, 5]. However, even though necrosis can be diagnosed reliably by imaging after local tumor ablation, hypoperfusion of tumor nodules induced by targeted therapies with antiangiogenic effects could be misleading and may falsely diagnose necrosis [14].

Only recently, the iRECIST guidelines were developed and proposed to be used in clinical trials testing immunotherapeutics, as response to immunotherapy may differ from that observed with other anticancer agents (e.g., late response, pseudoprogression; [15]). Table 1 summarizes the definition of CR according to RECIST and its subsequent modifications over the past years.

**Table 1:** Definition of complete response

Criteria (year)	Definition
RECIST (2000)	Disappearance of all target and non-target lesions; normalization of tumor marker levels
RECIST 1.1 (2009)	Disappearance of all target and non-target lesions; short axis of all lymph nodes <10 mm; normalization of tumor marker levels
mRECIST (2010)	Disappearance of any intratumoral arterial enhancement in target and non-target lesions
iRECIST (2017)	Disappearance of all target and non-target lesions; short axis of all lymph nodes <10 mm

*iRECIST* modified Response Evaluation Criteria in Solid Tumors version 1.1 for immune-based therapeutics; *mRECIST* modified Response Evaluation Criteria in Solid Tumors; *RECIST* Response Evaluation Criteria in Solid Tumors; *RECIST 1.1* Response Evaluation Criteria in Solid Tumors version 1.1

Notably, CR does not necessarily mean that the cancer is cured. According to Easson and Russel, the cure of a disease means that after a certain time period, there are some disease-free survivors that have an all cause-mortality comparable to that of the sex- and age-matched general population [16]. Although it is difficult to find a widely accepted definition and it certainly varies between tumor types, most oncologist would use the term “cure” after a period of more than 5 years of remission [17].

## Liver cirrhosis predisposes for recurrence

The majority of HCC patients suffers from underlying liver cirrhosis which represents the main risk factor for the development of HCC [2, 18]. About 1–8% of patients with cirrhosis develop HCC yearly [19]. Even though successful treatment of the underlying liver disease (e.g., hepatitis C) decreases HCC risk in patients with cirrhosis [20], a substantial risk remains [21, 22]. Hence, liver cirrhosis hampers cure even in very early and potentially curable tumor stages as patients with cirrhosis can develop *de novo* HCC independently of the primary tumor. Generally, late recurrence is often referred to as *de novo* tumors, while true recurrence due to dissemination usually occurs earlier after treatment [23]. About 64–77% of patients treated with curative intent by surgery or local ablation experience recurrence at five years [24].

Liver transplantation is the only treatment that can cure both the tumor and underlying cirrhosis, and therefore recurrence in the transplantation setting mainly depends on tumor burden and biology at the time of transplantation. Hence, only if strict selection criteria (small tumors, no MVI, no extrahepatic metastases) are applied can transplantation achieve excellent survival and recurrence rates in patients with HCC (overall and recurrence-free survival rates at 4 years, 85 and 92%; [25]).

## Complete remission with systemic therapy

### Targeted therapies

Targeted therapies represent the mainstay in the treatment of patients with advanced/metastatic HCC [4]. To date, the multi-tyrosine kinase inhibitors sorafenib in the first-line and regorafenib in second-line have been approved for HCC [4] and other targeted therapies—lenvatinib, cabozantinib, and ramucirumab—have demonstrated efficacy in phase III studies [6–8]. Unlike cytotoxic chemotherapy, these agents generally lead to disease stabilization rather than tumor shrinkage [26]. However, even though a rare event (≤1%), CR has been reported for some molecular targeted therapies tested in phase III studies (Table 2; [6–8, 27–38]). Most data are available on sorafenib, as this drug was approved as the first systemic treatment for HCC over a decade ago, while other agents became available only recently [4]. Several cases of complete response with sorafenib have been reported in the literature, of which almost all patients had advanced stage HCC characterized by macrovascular tumor invasion and/or extrahepatic disease [39–58].

**Table 2:** Complete response with targeted therapies in randomized phase III trials of advanced hepatocellular carcinoma

			Complete response according to	
Study (reference)	Compound (*N* of patients)	Radiological criteria used	Investigator assessment, *N* (%)	Central independent review, *N* (%)
SHARP [32]	Sorafenib (299)	RECIST	N/R	0
	Placebo (303)	RECIST	N/R	0
Asia-Pacific [28]	Sorafenib (150)	RECIST	N/R	0
	Placebo (76)	RECIST	N/R	0
SUN 1170 [29]	Sunitinib (530)	RECIST	2 (<1)	N/R
	Sorafenib (544)	RECIST	1 (<1)	N/R
BRISK-PS [31]	Brivanib (263)	mRECIST	N/R	0
	Placebo (132)	mRECIST	N/R	0
BRISK-FL [30]	Brivanib (577)	mRECIST	2 (<1)	N/R
	Sorafenib (578)	mRECIST	5 (1)	N/R
EVOLVE-1 [33]	Everolimus (362)	RECIST	0	N/R
	Placebo (184)	RECIST	0	N/R
LIGHT [27]	Linifanib (514)	RECIST 1.1	N/R	N/R
	Sorafenib (521)	RECIST 1.1	N/R	N/R
SEARCH [35]	Sorafenib + Erlotinib (362)	RECIST	2 (<1)	N/R
	Sorafenib (358)	RECIST	1 (<1)	N/R
REACH [34]	Ramucirumab (283)	RECIST 1.1	1 (<1)	N/R
	Placebo (282)	RECIST 1.1	0	N/R
RESORCE [36]	Regorafenib (379)	mRECIST/RECIST 1.1	2 (1%)/0	N/R
	Placebo (194)	mRECIST/RECIST 1.1	0/0	N/R
REFLECT [6]	Lenvatinib (478)	mRECIST/RECIST 1.1	6 (1)/−	10 (2)/2 (<1)
	Sorafenib (476)	mRECIST/RECIST 1.1	2 (<1)/−	4 (1)/1 (<1)
METIV-HCC [38]	Tivantinib (226)	RECIST 1.1	N/R	0
	Placebo (114)	RECIST 1.1	N/R	0
JET-HCC [37]	Tivanitinib (134)	RECIST 1.1	N/R	N/R
	Placebo (61)	RECIST 1.1	N/R	N/R
CELESTIAL [8]	Cabozantinib (470)	RECIST 1.1	0	N/R
	Placebo (237)	RECIST 1.1	0	N/R
REACH-2 [7]	Ramucirumab	RECIST 1.1	0	N/R
	Placebo	RECIST 1.1	0	N/R

*mRECIST* modified Response Evaluation Criteria in Solid Tumors; *N/R* not reported; *RECIST* Response Evaluation Criteria in Solid Tumors; *RECIST 1.1* Response Evaluation Criteria in Solid Tumors version 1.1

In 7 of the phase III studies listed in Table 2, a total of 2926 patients received sorafenib monotherapy as test drug or comparator. Of these, only 9 (0.3%) patients achieved CR when assessed according to per protocol criteria. However, it must be noted that different response evaluation criteria were used (RECIST, RECIST 1.1, mRECIST) and radiological assessment was done locally in some trials and centrally in others.

In a nationwide Japanese case–control study, 18 of 3047 (0.6%) patients treated with sorafenib achieved a complete response according to mRECIST [59]. Three patients had portal vein invasion and 8 patients presented with distant metastases. The median time to CR was 119 days (range, 35–447 days). Female sex, low body weight, early clinical stage, and a low initial dose of sorafenib were more frequently observed in patients with CR. Furthermore, hand–foot–skin reaction, arterial hypertension, diarrhea, alopecia, fatigue, anorexia, and nausea occurred more often in complete responders [59]. Notably, the occurrence of early dermatological adverse events was independently associated with improved survival in a prospective study and may represent a surrogate marker for enhanced sorafenib efficacy [60].

Another retrospective multicenter study from Korea [61] reported that 7 of 523 patients (1.3%) treated with sorafenib achieved CR according to mRECIST after a median time of 3 months. All patients had advanced stage HCC, 6 due to vascular invasion and 1 because of bone metastasis. Recurrence was observed in 3 patients 3, 10, and 12 months after achieving CR; 2 patients discontinued sorafenib before or after experiencing CR and one patient continued sorafenib treatment. Median disease-free survival was only 9 months [61].

In a retrospective Spanish multicenter study [14], 12 of 1119 patients (1.07%) treated with sorafenib were classified as complete responders according to RECIST 1.1 plus SHARP trial criteria amendments (define additional nodules in cirrhosis, avoid registration of ascites and pleural effusion as progression; [62]). Ten patients had advanced HCC, 8 because of macrovascular invasion and 2 subjects due to extrahepatic spread (peritoneal and lung). Notably, two patients had very large tumors with a diameter of 7.5 and 11.0 cm, respectively. The median time to CR was 13.3 months (range, 0.9–33.3 months). The outcome was excellent with a median overall survival of 85.8 months (95%CI, 67.8–103.8 months). Four patients were still on sorafenib at the end of follow-up and did not show recurrence. Of 7 patients who discontinued sorafenib after experiencing complete response, 5 subjects had tumor recurrence and the other 2 remained in complete remission. Median time to recurrence after sorafenib discontinuation was 16.9 months (range, 8.5–73 months). All except of one patient—the only patient who initiated sorafenib at half dose—developed early dermatological side effects. The authors proposed that CR may result from sorafenib-mediated immune modulation [14].

### Immune checkpoint blockers

Checkpoint inhibitors have become a mainstay in the treatment of certain malignancies, including melanoma and lung cancer, and achieved excellent response rates in these tumor types [63, 64]. Until now, several phase I and II studies have investigated checkpoint blockers in advanced stage HCC, but despite promising overall response rates (up to around 25%), CR was a rare event [65–72] (Table 3). Interpretation of these data is limited by a small sample size in most studies and the lack of a control group.

**Table 3:** Complete response with immune checkpoint inhibitors in selected trials of advanced hepatocellular carcinoma

			Complete response according to	
Author, year (reference)	Compound (*N* of patients)	Radiological criteria used	Investigator assessment, *N* (%)	Central independent review, *N* (%)
*Monotherapy*				
Sangro, 2013 [69]	Tremelimumab (21)	RECIST	0	N/R
Crocenzi, 2016 [65]	Nivolumab (262)	RECIST 1.1	7 (2.7)	4 (1.5)
Wainberg, 2017 [70]	Durvalumab (40)	RECIST 1.1	0	N/R
Zhu, 2018 [66]	Pembrolizumab (104)	RECIST 1.1	N/R	1 (1)
*Combination with checkpoint inhibitors*				
Kelley, 2017 [71]	Durvalumab + tremelimumab (40)	RECIST 1.1	0	N/R
*Combination with ablation*				
Duffy, 2017 [72]	Tremelimumab + subtotal ablation (32)	RECIST 1.1	0	N/R
*Combination with targeted therapies*				
Ikeda, 2018 [67]	Lenvatinib + pembrolizumab (26)	mRECIST	1 (3.8)	N/R
Stein, 2018 [68]	Atezolizumab + bevacizumab (23)	RECIST 1.1	0	1 (4.3)

*mRECIST* modified Response Evaluation Criteria in Solid Tumors; *N/R* not reported; *RECIST* Response Evaluation Criteria in Solid Tumors; *RECIST 1.1* Response Evaluation Criteria in Solid Tumors version 1.1

The largest study published to date investigated nivolumab in a phase I/II dose-escalation/dose expansion study in sorafenib-naive (*n* = 80) and sorafenib-experienced (*n* = 182) patients [9, 65]. Of 262 patients in total, 7 (2.7%) had CR according to RECIST 1.1 by investigator assessment and 4 (1.5%) when evaluated by blinded central review [65]. In subgroup analysis, sorafenib-experienced patients with complete or partial response (*n* = 22) had an excellent outcome with survival rates at 18 months and 45 months of 100 and ∼90%, respectively [73]. Recently presented preliminary data from a single-arm phase II study testing pembrolizumab in sorafenib-experienced patients (*n* = 104) reported 1 CR (1%; [66]). Taken together, even though checkpoint blockers can induce durable responses in advanced HCC [9, 66], CR was reported only occasionally in phase I/II studies. Large phase III trials testing checkpoint inhibitors in advanced HCC are underway (e.g., nivolumab [NCT02576509], pembrolizumab [NCT02702401, NCT03062358], durvalumab/tremelimumab [NCT03298451], atezolizumab [NCT03434379]) and their results are eagerly awaited.

## Conclusion and future perspectives

CR in locally advanced or metastatic HCC is a possible but rare event. Achieving CR can be associated with long-term remission and translate into an excellent outcome. However, a substantial number of patients will experience tumor recurrence, which is partly owed to underlying liver cirrhosis.

Further efforts are necessary to gradually increase the proportion of patients with advanced HCC experiencing long-term remission. The combination of immunotherapy plus molecular targeted therapies with antiangiogenic effects seems reasonable, as antiangiogenics can induce tumor hypoxia [74] which in turn promotes immunosuppression, e.g., via upregulation of checkpoint molecules [75, 76]. Different combinations of targeted therapies and checkpoint blockers are currently investigated in several trials of advanced HCC [77]. Preliminary data from two pilot trials testing lenvatinib plus pembrolizumab [67] and the combination of atezolizumab (anti-PD-L1) and bevacizumab (anti-VEGF; [68]) are promising and demonstrated a complete response rate of 3.8 and 4.3%, respectively (Table 3). Strategies to reprogram the immunosuppressive tumor microenvironment towards an immunostimulatory milieu (e.g., renin–angiotensin inhibitors, depletion of fibrosis, anti-transforming-growth-factor-β) may have the potential to further enhance the efficacy of immunotherapy [76].

### Take home message

- Complete response to systemic therapy in locally advanced or metastatic HCC is a possible but rare event
- Achieving complete response can result in long-term remission and excellent outcome
